# Eco-friendly enhancement of potato micropropagation using bacterial-derived IAA from *Kocuria rosea*

**DOI:** 10.1038/s41598-026-67816-z

**Published:** 2026-09-04

**Authors:** Dina M. S. Hassan, Yasmin M. R. Abdellatif, Mohamed E. El-Demerdash, Enas A. Hassan

**Affiliations:** 1 Agricultural Microbiology Dept, Fac. of Agric, Ain Shams Univ., Cairo, Egypt; 2 Agricultural Botany Dept, Fac. of Agric, Ain Shams Univ, Cairo, Egypt

**Keywords:** Indoles, *Kocuria*, Bacterial supernatant, Spunta, Tissue culture, Biotechnology, Microbiology, Plant sciences

## Abstract

Indole-3-acetic acid (IAA), a key phytohormone widely synthesized by soil bacteria, plays a vital role in root development and stress resilience. This study aimed to optimize the culture conditions for IAA production by *Kocuria rosea* SS1 and to evaluate the efficacy of bacterial derived IAA on the micropropagation of potato (*Solanum tuberosum* L. cv. Spunta). Maximum IAA production (10.83 mg 100 mL^− 1^, as quantified by HPLC) was achieved in nutrient broth at pH 7 and 30 °C, supplemented with 1 mM tryptophan and incubated under shaking conditions for three days. Filter-sterilized bacterial supernatant containing IAA (0.0–1.0 mgL^− 1^) was incorporated into Murashige and Skoog (MS) medium and compared to commercial IAA in potato tissue culture. After 28 days, plantlets treated with bacterial IAA, particularly at 0.25 mgL^− 1^; showed significant enhancements in fresh weight (1.5-fold), roots number (69.9%), leaves number (100%) and shoot length (50%) compared to the control, outperforming equivalent doses of synthetic IAA. Biochemical analyses revealed increased levels of total sugars, chlorophylls, carotenoids, and endogenous IAA and gibberellins. These findings demonstrate that the bacterial supernatant serves as a potent, eco-friendly biostimulant that enhances the growth and biochemical quality of in vitro-propagated potato plantlets, offering a sustainable alternative to synthetic growth regulators.

## Introduction

Plant growth regulators (PGRs) are defined as synthetic or natural occurring organic compounds that influence biological processes in higher plants at very low concentrations. PGRs are categorized into several distinct classes, such as auxins (Aux), cytokinins (CKs), nitric oxide (NO), brassinosteroids (BRs), gibberellins (GAs), salicylic acid (SA), abscisic acid (ABA), jasmonates (JAs), and ethylene^1^. By origin, growth regulators are divided into the following groups: endogenous compounds synthesized by plants (phytohormones); products of microbial synthesis; and synthetic compounds. Because growth regulators of microbial origin are similar to compounds synthesized by plants (auxins, gibberellins, abscisic acid, cytokinins), they are also called phytohormones^2,3^.

Phytohormones are signal molecules, acting as chemical messengers that control plant growth and development, which is synthesized in trace amounts in certain parts of the plant and can be transported to other parts for the implementation of biochemical, physiological, and morphological responses, and also play major role in inducing tolerance to plants against various abiotic and biotic stresses by modulating the expression of stress-related genes^4^. Auxin is a widely used plant growth regulator of low molecular weight with an aromatic ring structure^5^. Auxin is produced in growing shoot tips and transported down the main stem *via* the polar auxin transport mechanism^6^. It is involved in cell division and elongation, organogenesis, and apical dominance^7^. Secondary metabolites synthesized by bacteria have become important in biotechnology^5^. One of the phytohormones produced by soil microorganisms is indole acetic acid (IAA)^8^. IAA production has been reported in various genera of PGPR, including *Acetobacter*, *Acinetobacter*, *Azospirillum*, *Arthrobacter*, *Azotobacter*, *Bacillus*, *Burkholderia*, Bradyrhizobium, *Mesorhizobium*, *Paenibacillus*, *Pseudomonas*, *Pantoea*, *Rhizobium*, *Rhodococcus*, *Serratia*, *Streptomyces*, *Strenotophomonas*, and *Rouxiella*^9^. Besides promoting plant growth, these microorganisms suppress soil-borne pathogens and contribute to PGPR-mediated induced systemic resistance (ISR), thereby enhancing plant defense, colonization, and overall plant^3^.

Potato (*Solanum tuberosum* L.) is a major global food crop, valued for its high carbohydrate, protein, vitamins, and mineral content, along with a low fat content. It can be arranged as the fourth most cultivated food crop after wheat, rice and maize^10^.

Vegetative propagation of potatoes *via* seed tubers results in the accumulation and spread of pathogens and pests such as viruses, bacteria, fungi, and nematodes. Up to 75% yield losses can be caused by viral infection alone^11^. More than 40 different viruses are known to infect cultivated potatoes^12,13^. Tissue culture is a simple powerful technique that allows researchers to grow and manipulate plants in a sterile environment; it is a useful method for plant breeding. Some of the many uses for plant tissue culture include rapid clonal propagation or micropropagation; disease elimination; anther/pollen culture to produce haploid plants…. etc. Kyte et al.^14^,, but potatoes are relatively insensitive to loss due to soil salinity, drought, and low nutrient availability^2^. Potato is the first major food crop in which biotechnology has been successfully implemented for seed production. Previous studies show that micropropagation of potatoes depends on the biological value of cultivars, explant type (leaf, node, shoot tip, etc.), type of culture medium, photoperiod, temperature, season, and a balanced combination of plant growth regulators (PGRs) in the culture media^15,16^.

This study aims to optimize IAA production by the bacterial strain *Kocuria rosea* SS1 and evaluate the impact of the bacterially derived IAA on potato micropropagation compared to commercial IAA.

## Results

### Optimization of culture conditions for IAA production by tested bacterium

**Fig. 1:**
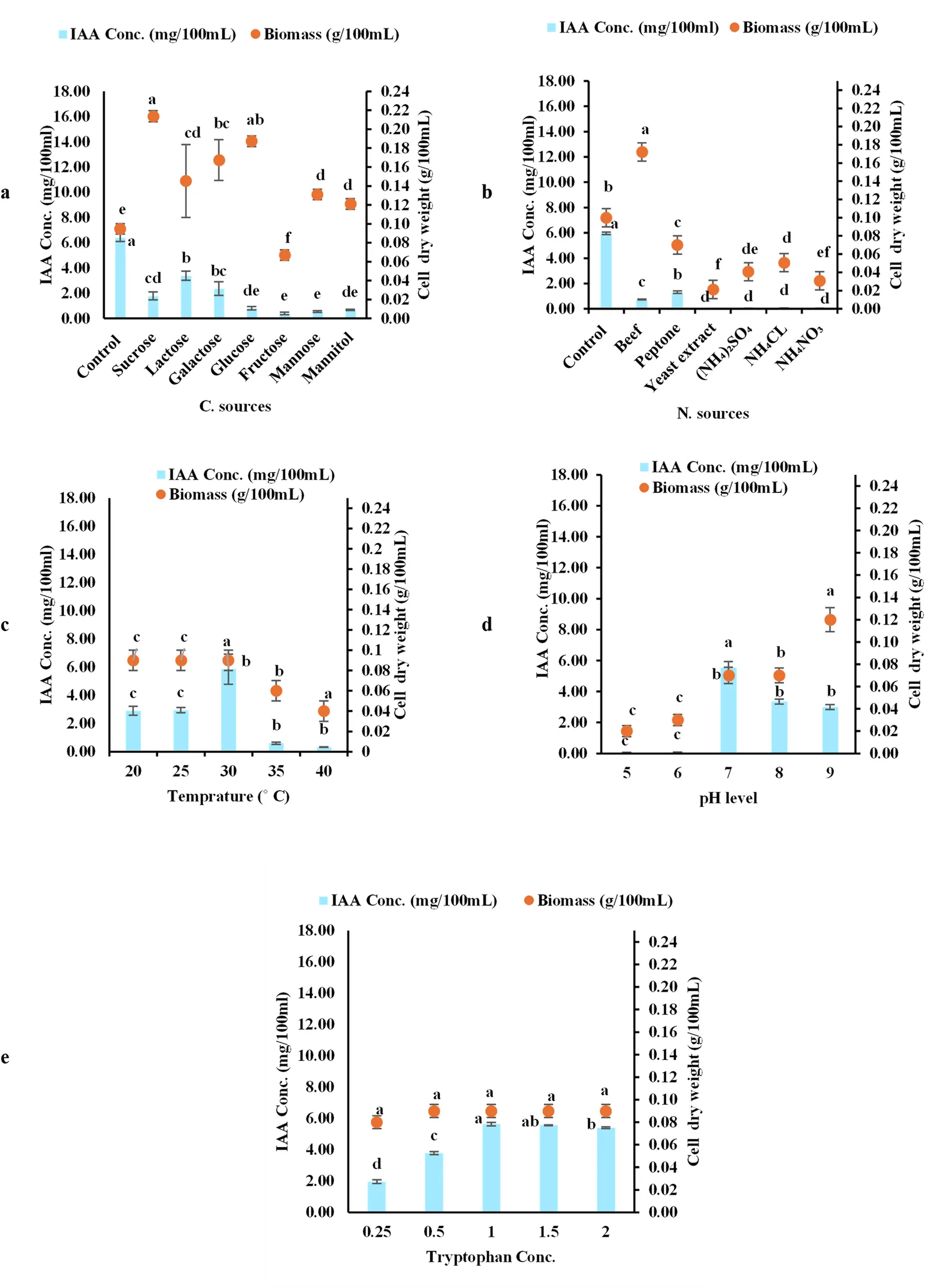
Effect of nutritional and environmental factors on IAA and biomass production by Kocuria rosea (SS1) strain grown in nutrient broth medium supplemented with L-tryptophan for 5 days under dark and shaking conditions. (**a**) different carbon sources, (**b**) different nitrogen sources, (**c**) different incubation temperatures and (**d**) initial pH medium (**e**) different concentrations of L-tryptophan. Each bar represents means ± SD of 3 replicate samples. Means followed by different letters are significantly different at *P* ≤ 0.05 level.

Carbon sources have been shown to positively affect phytohormone synthesis by supplying energy and improving cofactor recycling in cells^17^. To evaluate the most suitable carbon source for biomass and optimize IAA production, *Kocuria rosea* (SS1) was cultured in nutrient broth supplemented with individual carbon sources (glucose, fructose, galactose, sucrose, mannose, mannitol, lactose). The nutrient broth medium without additions achieved the highest indole-3-acetic acid (IAA) production, followed by lactose, with 6.39 and 3.39 mg IAA/100 mL, respectively. No correlation was observed between IAA production and cell dry weight. Glucose and sucrose were then used to optimize biomass dry weight, yielding 0.19 and 0.21 g/100 mL, and 0.81 and 1.79 mg/100 mL for IAA production, respectively. Fructose, mannose, and mannitol yielded the lowest IAA productivity and cell dry weight (Fig. 1a). Nitrogen sources were investigated as organic and inorganic forms. In general, supplementation with organic sources (beef extract, peptone) stimulated biomass dry weight and IAA production in *Kocuria rosea* (SS1) compared with inorganic nitrogen sources (ammonium sulphate, ammonium chloride, ammonium nitrate), which had a negative effect (Fig. 1b). The nutrient broth medium with its original components (beef + peptone) achieved the highest IAA productivity, giving 5.97 mg IAA/100 mL. *Kocuria rosea* (SS1) was cultivated in nutrient broth medium, which contains its carbon and nitrogen sources, as a static culture or in a shake flask agitated at 150 rpm. The data indicated that shaking increased cell dry weight and indole production by about 1–4-fold and 9-fold, respectively.

Data presented in Fig. (1c) show a significant increase in IAA production with increasing the incubation temperature from 25 to 30 ° C. Whereas the cell dry weight recorded no significant difference between the values at 20, 25 and 30 ° C. The optimum temperature for cell dry weight and IAA production was 30 ° C, which gave 0.09 g/100 mL and 5.85 mg IAA/100 mL, respectively. It was observed that there was a sharp decrease in IAA production by increasing the incubation temperature.

The data of cell dry weight and IAA production by *Kocuria rosea* (SS1) as influenced by initial pH values were recorded in Fig. (1d). The results indicated that pH 7 was the most favorable for IAA production by *Kocuria rosea* (SS1), giving 5.53 mg/100 mL. Figure (1e) revealed the stimulatory effect of L-tryptophan concentration on IAA production by *Kocuria rosea* (SS1) up to 1.00 mM, giving 5.64 mg/100 mL, whereas higher concentrations had no significant effect on IAA production. Supplementation with different concentrations of L-tryptophan in the growth medium had no significant effect on cell dry weight.

### Time course of IAA production

**Fig. 2:**
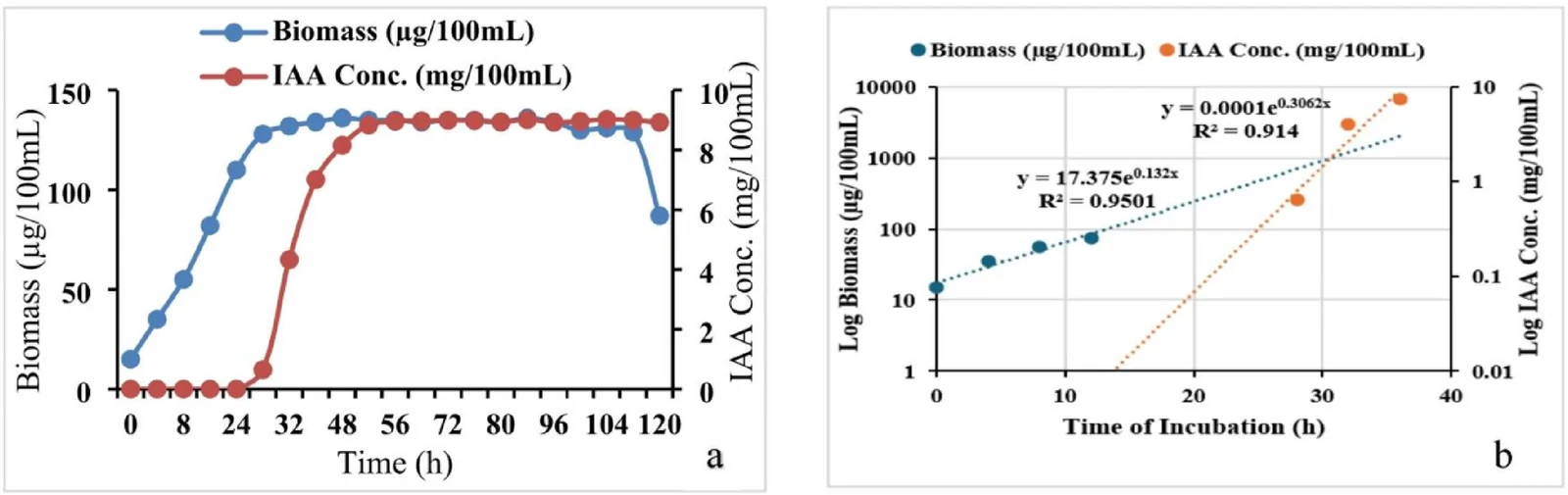
The fermentation time for IAA production by *Kocuria rosea* (SS1) strain grown in nutritional optimal conditions at 30˚C under shaking condition (**a**). The correlation coefficient between biomass or IAA and fermentation time (**b**).

Data given in Fig. (2a) show the relation between the fermentation time and biomass &IAA produced by *Kocuria rosea* (SS1) grown in nutrient broth medium supplemented with 1 mM L-tryptophan at 30 ˚C under shaking conditions (150 rpm). It is obvious that the exponential phase of growth lasted for 28 h of the incubation period, and IAA production started with the stationary phase and achieved the maximum production values after 72 h (8.99 mg/100 mL). Figure (2b) presents a positive correlation coefficient between the cell dry weight and time of incubation (h) with an R2 value of 0.9501. As well as a positive correlation coefficient between the IAA production and fermentation time (h), with an R2 value of 0.9140.

### Quantification of IAA production by HPLC

*Kocuria rosea* SS1 was cultivated under the optimal nutritional and environmental conditions described previously. HPLC analysis of the culture supernatant revealed an indole-3-acetic acid (IAA) concentration of **10.83 mg/100 mL**. The culture supernatant was subsequently used as a natural source of IAA to enhance the in vitro micropropagation of potato **(*Solanum tuberosum*** **L. cv. Spunta).**

### Effect of different concentrations of IAA supernatant or commercial IAA on growth parameters of potato plantlets (*Solanum tuberosum* L., Spunta) in vitro

**Fig. 3:**
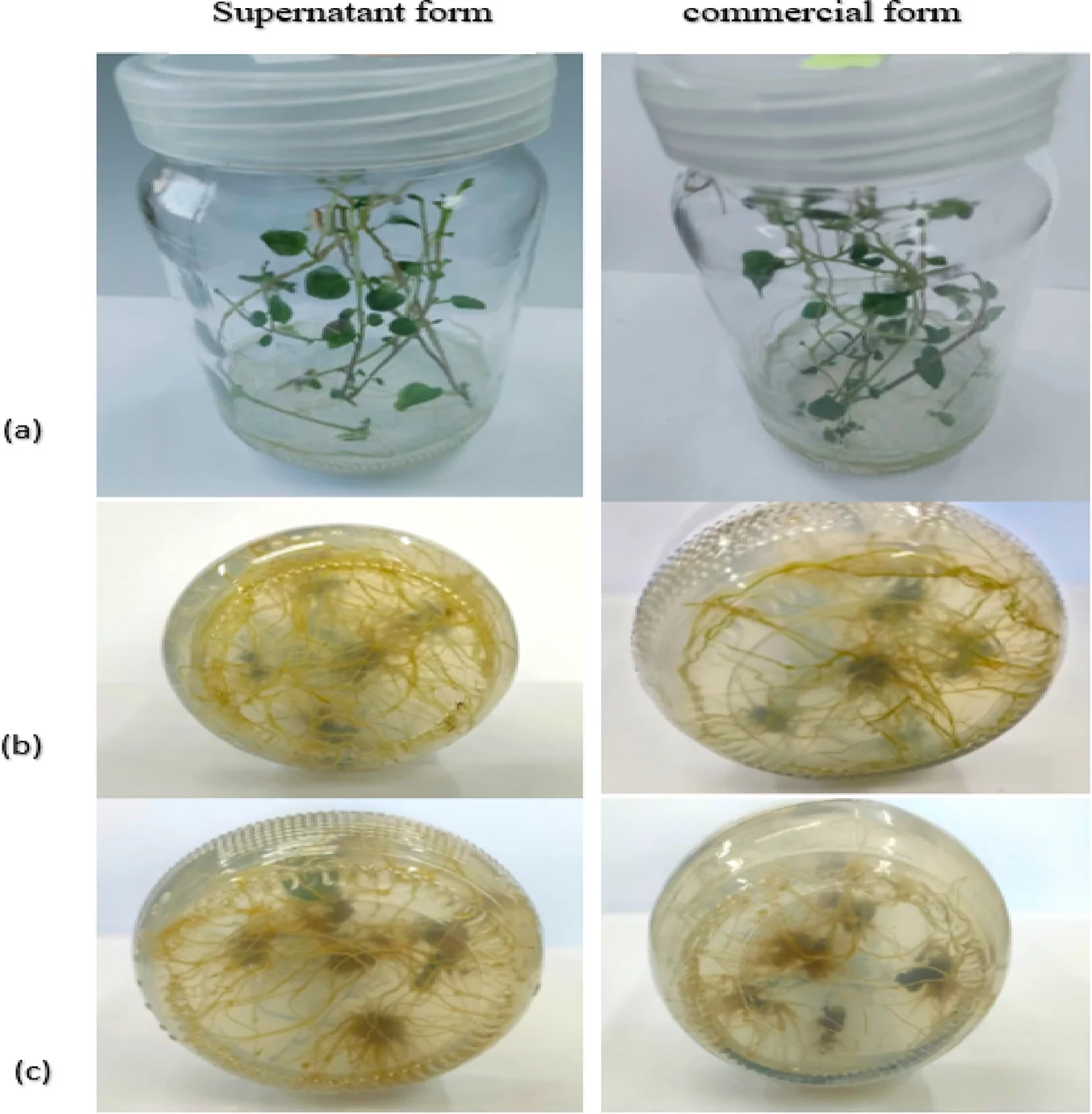
Effect of different concentrations of supernatant containing IAA or commercial IAA on potato plantlets at concentrations 0.25 mg/L (**a**), 0.50 mg/L (**b**), and 1.00 mg/L (**c**), after 4 weeks.

Tables 1 and 2 show that plantlets cultured on a medium supplemented with IAA supernatant exhibited higher growth parameters than those treated with commercial IAA. These increases were significant for plantlet fresh weight, root number, leaf number, and root length per plantlet. An insignificant difference was observed in shoot length between the two forms of IAA. Overall, the medium fortified with different concentrations of IAA treatments enhanced the growth parameters of potato plantlets compared to untreated plantlets. The highest concentration of IAA at 1 mg L^-1^ led to increases in most recorded growth measurements. However, there were no significant differences between IAA at 1 mg L^-1^ and IAA at 0.25 mg L^-1^ in plantlet fresh weight, leaf number, shoot length, and root length per plantlet. When comparing the interaction between both forms of IAA (supernatant or commercial IAA) across different concentrations, IAA supernatant at 0.25 mg L-1 produced the greatest increases in plantlet fresh weight (1.5-fold), root number (69.9%), leaf number (2-fold), and shoot length (0.5-fold) per plantlet. Insignificant differences were recorded in plantlet root length at IAA concentrations of 0.25 mg L^-1^ and 0.10 mg L^-1^ (Fig. 3). The lowest values for plantlet FW, root number, and root length per plantlet were recorded in control plantlets compared to plantlets treated with either IAA supernatant or commercial IAA at different concentrations.

**Table 1:** Effect of different concentrations of IAA supernatant or commercial IAA on fresh weight & roots and leaves numbers/potato plantlets (*Solanum tuberosum* L., Spunta) in vitro after 28 days of culturing.

IAA concentrations (mgL^− 1^)	Plantlet FW (g)			Roots number/plantlet			Leaves number/Plantlet		
	Natural	Commercial	means	Natural	Commercial	means	Natural	Commercial	means
0.00	0.53 **±** 0.04^e^	0.53 **±** 0.04^e^	0.53^C^	5.33 **±** 1.15^e^	5.33 **±** 1.15^e^	5.33^C^	10.33 **±** 0.58^bcd^	10.33 **±** 0.58^bcd^	10.33^A^
0.10	0.79 **±** 0.15^bcd^	0.67 **±** 0.16^cde^	0.73^B^	9.33 **±** 0.58^cd^	7.33 **±** 2.31^de^	8.33^B^	13.00 **±** 1.73^ab^	8.33 **±** 0.58^cd^	10.67^A^
0.25	1.50 **±** 0.20^a^	0.58 **±** 0.03^ed^	1.03^A^	11.33 **±** 1.15^bc^	6.67 **±** 1.15^de^	9.00^B^	15.00 **±** 3.00^a^	7.33 **±** 0.58^d^	11.17^A^
0.50	0.90 **±** 0.22^bc^	0.83 **±** 0.05^bc^	0.87^B^	14.00 **±** 1.73^ab^	12.67 **±** 2.52^ab^	13.33^A^	10.67 **±** 1.15^bc^	9.67 **±** 2.08^cd^	10.17^A^
1.00	1.04 **±** 0.21^b^	1.02 **±** 0.05^b^	1.03^A^	15.33 **±** 2.89^a^	13.33 **±** 1.53^ab^	14.33^A^	13.00 **±** 1.15^ab^	10.00 **±** 2.00^bcd^	11.50^A^
Means	0.95^A^	0.72^B^		11.07^A^	9.07^B^		12.40^A^	9.13^B^	

Each value followed by different letters are significantly different at *P* ≤ 0.05 level; Tukey’s Studentized Range (HSD) test, capital letters for means of natural- commercial growth regulators (horizontal row) or different concentration of plant growth regulators (vertical columns), whereas lowercase letters for interaction.

**Table 2:** Effect of different concentrations of IAA supernatant or commercial IAA on shoot and root length/plantlet of potato plantlets (*Solanum tuberosum* L., Spunta) in vitro after 28 days of culturing.

IAA concentrations (mgL^− 1^)	Shoot length (cm)/plantlet			Root length (cm)/plantlet		
	Natural	Commercial	means	Natural	Commercial	means
0.00	12.63 ± 2.20^abc^	12.63 ± 2.20^abc^	12.63^A^	11.33 ± 1.53^c^	11.33 ± 1.53^c^	11.33^B^
0.10	10.27 ± 0.75^cd^	11.77 ± 2.05^bcd^	11.017^AB^	15.50 ± 0.87^a^	12.60 ± 1.83^abc^	14.05^A^
0.25	15.83 ± 2.36^a^	10.67 ± 0.29^cd^	13.25^A^	14.27 ± 1.45^abc^	13.33 ± 2.08^abc^	13.80^A^
0.50	8.83 ± 0.58^d^	11.50 ± 1.32^bcd^	10.17^B^	14.73 ± 2.41^a^	13.17 ± 0.76^abc^	13.95^A^
1.00	14.50 ± 2.18^ab^	11.90 ± 1.77^bcd^	13.20^A^	13.67 ± 2.08^abc^	12.30 ± 1.61^bc^	12.98^AB^
Means	12.41^A^	11.69^A^		13.90^A^	12.55^B^	

Each value followed by different letters are significantly different at *P* ≤ 0.05 level; Tukey’s Studentized Range (HSD) test, capital letters for means of natural- commercial growth regulators (horizontal row) or different concentration of plant growth regulators (vertical columns), whereas lowercase letters for interaction.

### Effect of different concentrations of IAA supernatant or commercial IAA on biochemical parameters of potato plantlets (*Solanum tuberosum* L., Spunta) in vitro

Insignificant differences were recorded between the supernatant and commercial IAA for total sugar, total soluble phenols, and carotenoids (Tables 3 and 4, and 5). The commercial IAA enhanced total amino acids, IAA, GA_3_ content, and total chlorophylls of the potato plantlet compared with the natural IAA-supplemented medium. Supernatant or commercial IAA elevated the concentrations of total sugar, IAA, GA_3_, total chlorophylls, and carotenoids compared to a medium devoid of IAA (control). Total sugars, total amino acids, IAA, GA_3_ content, and chlorophylls increased with both natural and commercial IAA at 0.1 mgL^-1^ compared to the control treatment (without IAA). The highest values of total amino acids (19.76 mg/g FW) and IAA content (0.72 mg/g FW) were recorded when commercial IAA was added at 0.1 mgL^-1^, whereas the lowest values of soluble phenols (6.51 mg/g FW) and total amino acids (7.56 mg/g FW) were recorded when natural IAA was added at 1 mgL^-1^ and 0.25 mgL^-1^, respectively. The natural IAA gave the highest value of total sugars at 0.1 mgL^-1^.

**Table 3:** Effect of different concentrations of IAA supernatant or commercial IAA on total sugars, total phenols and total amino acids of potato plantlets (*Solanum tuberosum* L., Spunta) in vitro after 28 days of culturing.

IAA concentrations (mgL^− 1^)	Total sugars (mg/g FW)			Total soluble phenols (mg/g FW)			Total amino acids (mg/g FW)		
	Natural	Commercial	means	Natural	Commercial	means	Natural	Commercial	means
0.00	2.81 ± 0.06^g^	2.81 ± 0.06^g^	2.81^E^	8.49 ± 0.20^ab^	8.49 ± 0.20^ab^	8.49^A^	14.80 ± 0.44^e^	14.80 ± 0.44^e^	14.79^B^
0.10	8.68 ± 0.16^a^	6.55 ± 0.21^b^	7.61^A^	8.42 ± 0.30^ab^	8.66 ± 0.40^ab^	8.54^A^	`	19.76 ± 0.24^a^	18.04^A^
0.25	4.55 ± 0.16^e^	6.43 ± 0.13^b^	5.49^B^	8.09 ± 0.08^b^	8.49 ± 0.10^ab^	8.29^A^	7.56 ± 0.03^h^	17.91 ± 0.05^b^	12.73^D^
0.50	5.00 ± 0.28^d^	5.56 ± 0.09^c^	5.28^C^	8.47 ± 0.30^ab^	5.78 ± 0.30^d^	7.13^C^	8.75 ± 0.12^g^	16.05 ± 0.20^d^	12.40^E^
1.00	4.52 ± 0.12^e^	4.01 ± 0.07^f^	4.26^D^	6.51 ± 0.30^c^	8.79 ± 0.20^a^	7.65^B^	11.07 ± 0.06^f^	16.67 ± 0.06^c^	13.87^C^
Means	5.11^A^	5.07^A^		7.99^A^	8.04^A^		11.70^B^	17.03^A^	

Each value followed by different letters are significantly different at *P* ≤ 0.05 level; Tukey’s Studentized Range (HSD) test, capital letters for means of natural- commercial growth regulators (horizontal row) or different concentration of plant growth regulators (vertical columns), whereas lowercase letters for interaction.

**Table 4:** Effect of different concentrations of IAA supernatant or commercial IAA on IAA and GA_3_ content of potato plantlets (*Solanum tuberosum* L., Spunta) in vitro after 28 days of culturing.

IAA concentrations (mgL^− 1^)	IAA (mg/g FW)			GA_3_ (mg/g FW)		
	Natural	Commercial	means	Natural	Commercial	means
0.00	0.38 ± 0.01^d^	0.38 ± 0.01^d^	0.38^C^	3.01 ± 0.06^f^	3.01 ± 0.06^f^	3.01^E^
0.10	0.46 ± 0.01^c^	0.72 ± 0.04^a^	0.59^A^	4.56 ± 0.18^d^	5.97 ± 0.27^c^	5.26^B^
0.25	0.23 ± 0.02^f^	0.74 ± 0.03^a^	0.48^B^	1.87 ± 0.03^g^	6.57 ± 0.18^b^	4.22^D^
0.50	0.36 ± 0.01^d^	0.59 ± 0.03^b^	0.48^B^	3.89 ± 0.09^e^	5.79 ± 0.23^c^	4.84^C^
1.00	0.32 ± 0.03^e^	0.31 ± 0.02^f^	0.32^D^	3.86 ± 0.07^e^	7.85 ± 0.28^a^	5.86^A^
Means	0.35^B^	0.55^A^		3.44^B^	5.84^A^	

Each value followed by different letters are significantly different at *P* ≤ 0.05 level; Tukey’s Studentized Range (HSD) test, capital letters for means of natural- commercial growth regulators (horizontal row) or different concentration of plant growth regulators (vertical columns), whereas lowercase letters for interaction.

**Table 5:** Effect of different concentrations of IAA supernatant or Commercial IAA on total chlorophylls and carotenoids of potato plantlets (*Solanum tuberosum* L., Spunta) in vitro after 28 days of culturing.

IAA concentrations (mgL^− 1^)	Total chlorophylls (mg/g FW)			Carotenoids (mg/g FW)		
	Natural	Commercial	means	Natural	Commercial	means
0.00	2.18 ± 0.11^d^	2.18 ± 0.11^d^	2.18^ؤ^	0.61 ± 0.02^de^	0.61 ± 0.02^de^	0.61^C^
0.10	2.57 ± 0.14^c^	3.39 ± 0.12^b^	2.98^B^	0.58 ± 0.01^ef^	0.67 ± 0.03^bc^	0.63^BC^
0.25	3.25 ± 0.16^b^	3.72 ± 0.08^a^	3.49^A^	0.74 ± 0.04^a^	0.68 ± 0.03^bc^	0.71^A^
0.50	2.51 ± 0.13^c^	3.23 ± 0.16^b^	2.87^B^	0.64 ± 0.03^cd^	0.74 ± 0.03^a^	0.69^AB^
1.00	2.61 ± 0.13^c^	1.92 ± 0.10^d^	2.27^C^	0.70 ± 0.01^ab^	0.59 ± 0.07^f^	0.65^B^
Means	2.64^B^	2.88^A^		0.65^A^	0.65^A^	

Each value followed by different letters are significantly different at *P* ≤ 0.05 level; Tukey’s Studentized Range (HSD) test, capital letters for means of natural- commercial growth regulators (horizontal row) or different concentration of plant growth regulators (vertical columns), whereas lowercase letters for interaction.

## Discussion

The IAA production was strongly influenced by biochemical characteristics and environmental factors**(**^18^. The accumulation of metabolic products was influenced by the media components (C, N, and growth factors)^19^. Carbon sources positively influenced IAA synthesis, likely because they supply energy and enhance cofactor recycling in cells^20^. In this study, the highest values of IAA production were achieved by *K. rosea* in nutrient broth medium (with the original components of carbon and nitrogen sources) supplemented with 1mM L-tryptophan at pH 7, incubated at 30 ˚C under dark and shaking conditions (150 rpm) for 3 days to record 8.99 mg/100 mL. These findings are like those of previous research in which the maximum IAA production was observed at pH 7. Also, many authors state that the optimum pH for IAA production is between 6 and 11^21^. The *K. rosea* strain was a mesophilic bacterium because optimal growth was observed at 28˚C under aerobic conditions. The temperature range for growth of *K. rosea* ranges between 25 and 37˚C, and the optimal growth was at 30˚ C. *Kocuria* sp. produced 46 µg/mL IAA when supplemented with a 600 µg/mL concentration of L-tryptophan at 96 h, which showed that IAA production was directly proportional to L-tryptophan^22^. Maximum production of auxin by the bacterial strains was during the stationary growth phase of bacterial isolates^23^. Kiruthika and Arunkumar^24^, reported that different concentrations of L-tryptophan were used to assess the induced effect on IAA production under optimized carbon and nitrogen sources. The shaking condition was found to have a remarkable impact on the production of IAA by *Providencia* sp. In the shaking condition, ~ 1.88-fold increase in IAA production (171.29 ± 0.10 µg/mL) was recorded compared to the static condition (90.65 ± 7.69 µg/mL). The shaking condition may have favored IAA production because of a good oxygen transfer rate, which makes the production medium homogeneous and increases the availability of biomass and oxygen, affecting the activity of enzymatic transformations from tryptophan to auxins^25^.

The optimization procedure in this study followed the one-factor-at-a-time (OFAT) approach, which identified the influence of individual variables on bacterial IAA production. However, because this approach does not consider possible interactions among factors, it may identify local rather than global optimum conditions. Therefore, future studies using multivariate optimization techniques, such as Response Surface Methodology (RSM), may further improve IAA production and maximize its plant growth-promoting effects.

Bacterial-derived phytohormones can be used to improve the efficiency and success rates of plant tissue culture, offering a sustainable and effective approach to plant propagation and growth enhancement. Phytohormones are involved in cell division, growth, adaptation, senescence, substance transport, respiration, and the synthesis of proteins and nucleic acids. However, each class of phytohormones exerts distinct effects on plant growth and development^26^. Similar to other plant species, potatoes possess a well-characterized complement of phytohormones. For instance, some phytohormones regulate different stages of plant growth, whereas others promote tuberization^27^. In plant cells, mitosis can be stimulated by high concentrations of auxins. In contrast, explant development is often slow in inexpensive, hormone-free media. However, the rate of explant development can be enhanced by supplementing the culture medium with natural phytohormones **(**^28,29^**)**.

Bacterial-derived phytohormones can be used to improve the efficiency and success rates of plant tissue culture, offering a sustainable and effective approach to plant propagation and growth enhancement. Phytohormones are involved in cell division, growth, adaptation, senescence, substance transport, respiration, and the synthesis of proteins and nucleic acids. However, each class of phytohormones exerts distinct effects on plant growth and development^26^. Similar to other plant species, potatoes possess a well-characterized complement of phytohormones. For instance, some phytohormones regulate different stages of plant growth, whereas others promote tuberization^27^. In plant cells, mitosis can be stimulated by high concentrations of auxins. In contrast, explant development is often slow in inexpensive, hormone-free media. However, the rate of explant development can be enhanced by supplementing the culture medium with natural phytohormones **(**^28,29^**)**.

Summarizing the growth parameters evaluated in this study, the greatest overall growth was observed in microplants cultured on the tested nutrient medium supplemented with IAA at concentrations of 0.1 and 0.25 mg L⁻¹. The biological responses observed in the present investigation should not be attributed solely to indole-3-acetic acid (IAA). Rather, the bacterial culture filtrate represents a complex biochemical matrix containing phytohormones in addition to a broad spectrum of bioactive metabolites produced during bacterial metabolism. Increasing evidence indicates that crude bacterial culture filtrates frequently exhibit greater plant growth-promoting efficacy than purified phytohormones because of the synergistic interactions among multiple microbial metabolites. These bioactive constituents may include siderophores, amino acids, vitamins, organic acids, exopolysaccharides, lipopeptides, volatile organic compounds, antioxidant metabolites, and various signaling molecules released during bacterial growth. Collectively, these compounds modulate numerous physiological and biochemical processes, thereby enhancing nutrient uptake, photosynthetic efficiency, antioxidant capacity, and plant resilience under adverse environmental conditions^30^.

These results are consistent with those reported by Stupko and Lugovtsova^31^, who found that the addition of IAA resulted in maximum growth of potato plantlets compared with plantlets cultured on an IAA-free medium. According to Sosnowski et al.^32^,, IAA promotes shoot and root growth and increases the number of lateral and adventitious roots. Growth processes have also been reported to be more strongly stimulated by exogenous auxins^33^. Ori et al.^34^, reported increased endogenous sugar levels in the roots, shoots, and leaves of *Phalaenopsis amabilis*, accompanied by an increase in endogenous auxin concentrations after 30 days.The most abundant auxin in plants, indole-3-acetic acid (IAA), is synthesized from its precursor, tryptophan, which is produced in the chloroplast. Auxins influence photosynthesis by affecting the structure and many essential components of the chloroplast (Salazar-Iribe and De-la-Peña 2020). Liu et al.^35^, reported that auxin is positively correlated with the expression of genes involved in chlorophyll biosynthesis. They further indicated that IAA enhances photosynthesis by stimulating chloroplast development and increasing photosynthetic activity, thereby leading to greater carbohydrate production. The decrease in free amino acid content observed in IAA-treated plantlets indicates enhanced nitrogen metabolism and overall metabolic activity. This response may be explained by the close relationship between auxin homeostasis and amino acid metabolism. The formation of IAA–amino acid conjugates represents an important regulatory mechanism linking auxin metabolism with amino acid biosynthesis, thereby contributing to the regulation of free amino acid pools in plant tissues^36^.

## Conclusion

In vitro, *Kocuria rosea* SS1 (OR944054) achieved the highest IAA production (10.83 mg/100 mL) when cultured in nutrient broth at pH 7 and 30 °C in the presence of 1 mM tryptophan for 3 days under shaking condition. Using the tissue culture technique, bacterial-derived indole-3-acetic acid (IAA) supplied as a culture supernatant at a concentration of 0.25 mg L⁻¹ in MS medium produced the greatest improvement in all growth parameters of potato plantlets. These findings highlight the potential of bacterial-derived IAA as a key component of sustainable agricultural practices. IAA-producing bacteria have demonstrated the ability to promote plant growth by enhancing water and nutrient uptake, particularly under adverse environmental conditions. Their capacity to produce IAA provides promising opportunities for biofertilization and bio-stimulation, offering an environmentally friendly alternative to chemical fertilizers while improving plant resilience to abiotic stresses. Furthermore, integrating bacterial culture filtrates into plant tissue culture protocols represents a promising biotechnological strategy for sustainable crop propagation. Future research should focus on elucidating the mechanisms underlying their growth-promoting effects, characterizing the complete spectrum of bioactive metabolites present in the bacterial culture filtrate, and evaluating their efficacy under greenhouse and field conditions.

## Materials and methods

### The bacterial culture used

The bacterium *Kocuria rosea*SS1 was isolated previously, from rhizosphere plant grown in Giza Government Egypt, recorded high quantity of IAA (6.30 mg/100mL) and identified biochemically and genetically by Hassan et al.^37^,. The sequences have been deposited at GenBank under Accession number OR944054. It was sub-cultured on nutrient agar and stored at 4 ˚C.

### Optimizing conditions for IAA production

Culture conditions were optimized to maximize IAA productionn by *Kocuria rosea *SS1. The carbon and nitrogen source, tryptophan concentration, incubation time, incubation temperature, pH medium were investigated. The Erlenmeyer flasks 250 mL containing 100 mL of nutrient broth medium was sterilized in autoclave at 121˚C/20min/1.2 atmospheric. Flasks were inoculated with 2 mL of a standard inoculum (10^7^ CFU/ml)of the test strain and incubated for 5–7 days under shaking condition (150 rpm). After incubated period, the broth culture was centrifuged at 11,180 × g for 10 min. The dry weight of biomass and IAA in clear supernatant were estimated. The indole production was determined using Salkowski’s reagent according to Gutierrez et al(2009). The concentration of indole in the supernatant was calculated from a standard curve (5–100 µg/ml) and expressed in mg/100 ml.

**Nutritional factors:** The experiment was conducted to study the effects of various carbon sources (glucose, fructose, galactose, sucrose, mannose, mannitol, lactose) and nitrogen sources (organic sources; beef extract, peptone, yeast extract& inorganic sources; ammonium sulphate, ammonium chloride, ammonium nitrate) on microbial phytohormones production. Different concentrations of L-tryptophan ranged from 0.25 to 2mM, were studied.

** Environmental factors:** The effect of different incubation temperatures (20- 25- 30- 35- 40 ° C), pH levels(ranged from 5 to 9), and incubation periods (at intervals each 4 h during 120 h of fermentation) on IAA production were assessed. As well as the static and shaking fermentation conditions were evaluated.

### High-performance liquid chromatography determination for IAA in bacterial culture

HPLC analysis of IAA was carried out using the condition of a UV detector set at 254 nm wavelength. Agilent 1100 Series HPLC system (Agilent Technologies, Santa Clara, CA, USA) equipped with a diode array detector (DAD). Separation was achieved on a C18 reversed-phase column. The mobile phase used was 0.1% acetic acid with acetonitrile 40:60 v: v at a flow rate of 1.5 mL/min. Samples and standard solutions were degassed and filtered through a 0.22 μm membrane filter. The sample injection volume was 15 µl. The retention time of sample peaks was compared with this of pure IAA standard in the Central Laboratory Unit Faculty of Science, Ain Shams University^38^.

### Potato plant propagation

Plant propagation experiment was conducted in Plant Tissue Culture Laboratory of Agricultural Botany Department, Faculty of Agriculture, Ain Shams University, Egypt, during the successive years of 2021, 2022 and 2023 to study the effect of different concentrations of IAA bacterial phytohormones obtained from filtrated-sterilized bacterial supernatant of selected bacterium on plant propagation by tissue culture technique.

### Plant material and sprout propagation

Spunta cultivar of certified virus free potato tubers (*Solanum tuberosum* L) were obtained from Strawberry Center, Faculty of Agriculture, Ain Shams University, Egypt. The tubers were washed under running water for 15 min, brushed gently, sprayed with distilled water, and stored in dark conditions to accelerate sprouting. Shoot tips (3–4 mm in length) were excised and surface-sterilized in the laminar flow by hydrogen peroxide (12% v/v) for 20 min^39^. A few drops of tween 20 were added. Meristematic tips and sup-tending leaf primordial (0.3–0.5 mm) were separated in aseptic condition and cultured in 100 ml jar (3 explants/jar) containing 20 ml of Murashige and Skoog (MS) (basal medium) according to Murashige and Skoog^40^. Jars were incubated directly after culturing in the incubation room at 25 ± 2 °C under 16 h photoperiod (2500 lx illumination) for six weeks.

### Micropropagation of nodal explants

Plantlets of Spunta cultivar were multiplied as single node cuttings with one leaf in 250.0 mL jar (5 explants/jar) containing 30.0 mL solidified MS medium supplemented with α naphthalene acetic acid (NAA) at 0.1 mg L^−1^, gibberellic acid (GA_3_) at 0.1 mg L^−1^ and benzyl adenine (BA) at 0.5 mg L^−1^^41,42^. After four weeks, plantlets with 6–7 nodes were used for subculturing with the same composition and concentrations of plant growth regulators of freshly prepared MS. Subculturing was re-cultured three times until the required number of plantlets was achieved.

### IAA Treatments on potato explant in vitro

The bacterial supernatant containing IAA was sterilized by filtration technique using millipore filter (0.22 μm) and used at different concentrations to study their effects on plantlets growth and multiplication. Two groups of MS medium were prepared. The filtrated supernatant at different concentrations; 0.00, 0.10, 0.25, 0.50, 1.00 mgL^−1^ for IAA were added separately into MS medium comparing to commercial IAA at the same concentrations^43^.

All the explants were incubated in controlled growth room at 25 °C ± 2 and 16 h photoperiod 2500 lx illumination for four weeks. After this incubation period, the plantlets were collected for both morphological observations and biochemical analyses.

#### Morphological parameters

Plantlet fresh weight (g), roots number/plantlet, leaves number/plantlet, shoot length (cm)/plantlet and roots length (cm)/plantlet were measured after four-weeks of incubation period.

#### Biochemical analyses

All biochemical analyses were performed on 0.2 g of fresh plant tissue as follows:

### Total sugars (TS)

Total sugars were quantified using the 3,5-dinitrosalicylic acid (DNSA) method according to the procedure of Krivorotova and Sereikaite^44^ and calculated from the calibration curve of standard D-glucose (5–1000 µg/mL), and the results were expressed as mg D-glucose/g fresh weight sample. The results were expressed as mg D-glucose/g fresh weight sample.

### Total soluble phenolic compounds (TSPC)

TSPC concentration of the different plant extracts was determined using Folin–Ciocalteu reagent, following the method of Singleton and Rossi^45^. The total soluble phenols concentration was calculated from the calibration curve of standard gallic acid (10–100 µl/ml) and expressed as mg gallic acid/g fresh weight.

### Total amino acids

Total amino acids concentration was determined using ninhydrin reagent. The developed purple color was measured using a spectrophotometer at 570 nm^46^. The total soluble amino acids concentration was calculated from the calibration curve of standard glycine (5–100 µl/ml) and expressed as mg amino acid/g fresh weight.

### Indole acitic acid and Gibberellic acid determination

Two milliliters of leaves extract was added to 2.00 mL of Salkowski reagent to determine IAA according to Glickman and Dessaux, (1995). The gibberellins were estimated spectrophotometric by standard method^47,48^in leaves extract^49–51^.

### Total chlorophylls and carotenoids concentrations

Accurately weighted 0.2 g of fresh plant leaf sample was taken and homogenized with 10 ml of 95% ethanol solvent. The solution mixture was analyzed for Chlorophyll-a, chlorophyll-b and carotenoids concentrations according to the method of Sumanta et al. (2014) The spectral absorbance for Chlorophyll-a, Chlorophyll-b, and carotenoids for ethanol solvent are represented in the following equations:

Ch-a = 13.36A664–5.19 A649.

Ch-b = 27.43A649–8.12 A664.

Cx + c= (1000A470 − 2.13Ca- – 97.63Cb)/209.

Chlorophyll-a (Ch-a), chlorophyll-b (Ch-b), carotenoids (Cx + c).

### Statistical analysis

The experiment was designed as a complete randomized design. The one-way ANOVA method and two-ways Anova method for interaction were employed using Lohr^52^**)** software. The means were calculated using three replicates, and Tukey’s Studentized Range (HSD) test was used to examine the significant differences at *p* ≤ 0.05 between the means.

## Data Availability

The authors can confirm that all data generated and analyzed during this study are included in this article.
